# No Effect of Anodal tDCS on Verbal Episodic Memory Performance and Neurotransmitter Levels in Young and Elderly Participants

**DOI:** 10.1155/2020/8896791

**Published:** 2020-09-22

**Authors:** Annegret Habich, Johannes Slotboom, Jessica Peter, Roland Wiest, Stefan Klöppel

**Affiliations:** ^1^University Hospital of Old Age Psychiatry and Psychotherapy, University of Bern, Bern, Switzerland; ^2^University Hospital of Psychiatry and Psychotherapy, University of Bern, Bern, Switzerland; ^3^Faculty of Biology, University of Freiburg, Freiburg, Germany; ^4^Support Center for Advanced Neuroimaging (SCAN), Neuroradiology, University Hospital of Bern, Inselspital, Bern, Switzerland; ^5^Institute of Diagnostic and Interventional Neuroradiology, University Hospital of Bern, Inselspital, Bern, Switzerland

## Abstract

Healthy ageing is accompanied by cognitive decline that affects episodic memory processes in particular. Studies showed that anodal transcranial direct current stimulation (tDCS) to the left dorsolateral prefrontal cortex (DLPFC) may counteract this cognitive deterioration by increasing excitability and inducing neuroplasticity in the targeted cortical region. While stimulation gains are more consistent in initial low performers, relying solely on behavioural measures to predict treatment benefits does not suffice for a reliable implementation of this method as a therapeutic option. Hence, an exploration of the underlying neurophysiological mechanisms regarding the differential stimulation effect is warranted. Glutamatergic metabolites (Glx) and *γ*-aminobutyric acid (GABA) are involved in learning and memory processes and can be influenced with tDCS; wherefore, they present themselves as potential biomarkers for tDCS-induced behavioural gains, which are affiliated with neuroplasticity processes. In the present randomized, double-blind, sham-controlled, crossover study, 33 healthy young and 22 elderly participants received anodal tDCS to their left DLPFC during the encoding phase of a verbal episodic memory task. Using MEGA-PRESS edited magnetic resonance spectroscopy (MRS), Glx and GABA levels were measured in the left DLPFC before and after the stimulation period. Further, we tested whether baseline performance and neurotransmitter levels predicted subsequent gains. No beneficial group effects of tDCS emerged in either verbal retrieval performances or neurotransmitter concentrations. Moreover, baseline performance levels did not predict stimulation-induced cognitive gains, nor did Glx or GABA levels. Nevertheless, exploratory analyses suggested a predictive value of the Glx : GABA ratio, with lower ratios at baseline indicating greater tDCS-related gains in delayed recall performance. This highlights the importance of further studies investigating neurophysiological mechanisms underlying previously observed stimulation-induced cognitive benefits and their respective interindividual heterogeneity.

## 1. Introduction

Whether you need to remember where you locked your bike, when you last called your sister, or whether you already gushed over the last episode of your favourite TV show to your coworker, episodic memory, the cognitive function that connects information with a specific context [1], is highly relevant for our daily routine. It is also one of the first cognitive functions affected by cognitive decline in healthy and pathological ageing; wherefore, counteractive therapeutic interventions are in high demand. Considering their involvement in the encoding and the retrieval of episodic memory contents [2, 3], the dorsolateral prefrontal cortices (DLPFC) present themselves as feasible targets for interventions. Among those, transcranial direct current stimulation (tDCS) modulates the excitability of neurons in the stimulated cortical area by inducing shifts in the membrane potential. Polarity-dependent tDCS effects are principally attributed to the polarisation of the soma of neurons radially aligned to the cortical surface [4, 5]. Somatic depolarisation leads to an increase in excitability whereas somatic hyperpolarisation results in a decrease in excitability making the cell more or less likely to fire action potentials [6]. Indeed, targeting the DLPFC with tDCS has previously facilitated behavioural performance in various episodic memory paradigms in healthy young [7–10] and healthy elderly adults [11, 12], individuals with subjective memory complaints [13], and patients with diagnosed mild cognitive impairment [14]. Despite the reported significant stimulation-induced benefits by single studies, a recent systematic meta-analysis confirmed this lack of unequivocal study results of tDCS in the episodic memory domain, reporting overall nonsignificant negligible effect sizes [15]. Partially, the discrepancies between study outcomes can be attributed to the different experimental designs (e.g., electrode parameters, stimulation intensity, duration, and behavioural task). Yet, abandoning the idea of consistent reliable group effects, a small set of studies also revealed one common factor that was repeatedly associated with more homogenous outcomes, namely, initial low performance in the tested task and the collateral room for improvement in this task. It is inconsequential whether this factor arises through suboptimal conditions in which the task is performed (e.g., in the morning for young participants [16]), greater task difficulty [17], or an intrinsic lower cognitive performance both in healthy young [18] and elderly adults [11, 19]. Even though effect sizes appear to be generally larger in both healthy [20] and cognitively impaired elderly [21], a reliable clinical application of this stimulation technique is not warranted yet. Rather than invoking a low behavioural performance as a marker for subsequent gain under stimulation, the underlying neurophysiological effects of the stimulation that ultimately lead to the observed improvements in behaviour need to be investigated.

By employing MEGA-PRESS-edited magnetic resonance spectroscopy (MRS), a noninvasive technique that allows the quantification of brain metabolites, multiple studies demonstrated that, in addition to sole cognitive effects of tDCS, the stimulation also affects *γ*-aminobutyric acid (GABA) and glutamatergic metabolites (Glx), the most abundant inhibitory and excitatory neurotransmitters in the brain, respectively. Consistent with neurotransmitter changes observed during learning and memory [22–24], the application of anodal tDCS led to a significant reduction of GABA in the sensorimotor area [25] as well as in the primary motor cortex [26–29], emphasizing its potential to produce neuroplastic changes in cortical areas. Furthermore, the reduction of the GABA level also correlated with the amount of motor learning [26, 28]. Conversely, anodal tDCS resulted in an increase in the glutamatergic tone in parietal regions [30, 31]. Taken together, this disinhibition, i.e., an elevated excitation-inhibition (E/I) ratio, supposedly facilitates the induction of long-term potentiation and thus allows for lasting effects beyond the period of online stimulation [32].

For prefrontal regions, however, evidence for the relationship between tDCS-induced neurophysiological and behavioural changes is still scarce and cannot be considered proven. Instead, given the different microstructure and physical properties of neurons in different brain areas [33], the effectivity of brain stimulation protocols across cortical regions can be called into question, and separate dose titrations are necessary for each cortical target. In order to close this gap, here, we tested the transferability of previous findings in the primary motor cortex to the DLPFC, adapting task, electrode setup, and location of the voxel, accordingly.

Based on the results of our preceding behavioural study [18], in which only initial low performers profited from anodal tDCS compared to sham stimulation, we aimed to replicate this differential effect of stimulation condition in healthy young and elderly adults, the latter of whom are known to perform worse in the chosen memory paradigm. In order to lessen the effects of interindividual variability, we switched from a parallel group study to a crossover design. To take a closer look at the neurophysiological underpinnings of tDCS-induced cognitive improvements, we extended the behavioural assessments by MRS spectrum acquisitions, with special focus on GABA and Glx, in the left DLPFC. Emulating the neurotransmitter changes reported in M1, we hypothesised that anodal tDCS will lead to a reduction of GABA and an elevation of Glx, in correspondence with an increase of cortical excitability described by the excitation-inhibition (E/I) ratio. Further, we expected that the change in neurotransmitter levels correlates with any behavioural gains. Additionally, we also probed whether baseline neurotransmitter levels predicted the subsequent neurotransmitter and behavioural changes and could be adduced as biomarkers for a stimulation-related gain.

## 2. Methods

### 2.1. Participants

After screening 56 young and 51 elderly adults for eligibility, 33 healthy young (aged 24.5 ± 2.6 years [mean ± SD], range: 20–30 years, 20 females) and 22 healthy elderly (aged 67.3 ± 4.4 years [mean ± SD], range: 60–74 years, 11 females) participants fulfilled all inclusion criteria and took part in the study (Table 1; for overview, see S1). Participants were right-handed (according to the Edinburgh Handedness Inventory, EDI; LQ > 70; [34]), native German speakers or possessed a comparable level of verbal intelligence (assessed via the German vocabulary test WST [35]), nonsmokers, had normal or corrected-to-normal vision, and had no history of psychiatric or neurological disorders. Likewise, participants with relevant depressive symptoms (according to Beck Depression Inventory II (BDI II) > 13; [36]) were excluded from the study. Healthy elderly required scores ≥ 19 in the Montreal Cognitive Assessment (MoCA; [37]) to participate in the study. Participants met all local tDCS and MRI safety criteria (i.e., no history of epileptic seizures, skin diseases, and/or magnetizable metal implants). Participants gave written informed consent prior to their participation in the study and were reimbursed with 100 CHF upon completion of all three sessions. The study was approved by the cantonal ethics commission of Bern (reference number: 2016-02175), registered at clinicaltrials.gov (identifier: NCT03227185), and conforms to the Declaration of Helsinki.

### 2.2. Study Procedure

**Figure 1:**
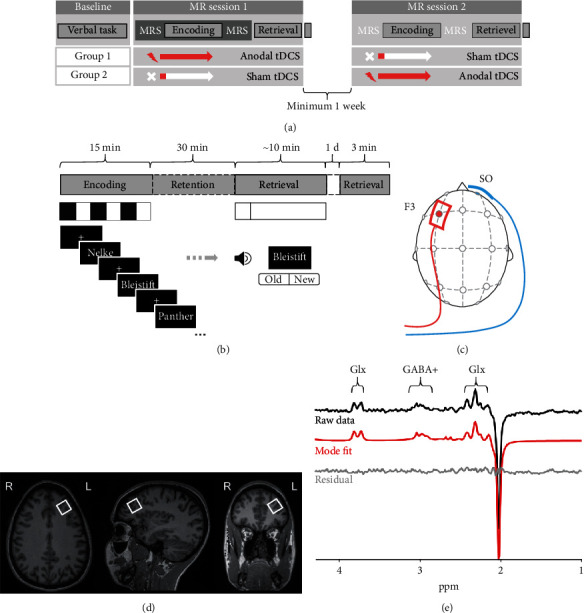
Experimental setup. (a) Study procedure during three experimental sessions. Baseline: assessments of initial cognitive task performance. MR sessions: verbal episodic memory task while receiving either sham or real anodal tDCS during encoding phase inside the MR scanner. MR spectra were acquired immediately before and after the encoding phase. Delayed retrieval outside MR scanner. Each participant received both sham and real anodal tDCS in a randomized sequence. (b) Verbal episodic memory task. The encoding phase consisted of presentation and immediate retrieval of forty nouns in three successive rounds. During the retrieval phase, participants performed a delayed free recall and a recognition task. Following the MR sessions, a second delayed recall was performed on the day after the session. (c) Electrode arrangement for application of anodal tDCS to the left DLPFC with the cathode placed supraorbitally on the contralateral side. (d) Example for voxel (2 × 2 × 2 cm) position in the left DLPFC. (e) Example of a 3T MR difference spectrum, including model fit and residuals.

In this double-blind, sham-controlled, crossover study, participants underwent three sessions on different days (Figure 1(a)). To avoid variability introduced by the circadian rhythm, sessions were restricted to the morning hours and participants came in at comparable times for each session. In the baseline session, participants familiarized themselves with the verbal learning task that consisted of three phases, namely, encoding, retention, and retrieval. On the following two study days, participants performed the same task with different words. The encoding phase took place in a 3T Siemens MAGNETOM Prisma MRI Scanner while participants received anodal tDCS over their left DLPFC. MRS data was acquired before and after the encoding phase. The delayed retrieval, consisting of a free recall and a recognition part, took place outside of the MRI scanner. After a consolidation night, another delayed recall was performed on the telephone. MR sessions were separated by at least one week to avoid carry-over effects of the stimulation.

To control for successful blinding, participants were asked to indicate the stimulation condition they thought they received at the end of the respective MR session. Additionally, participants completed a side effect questionnaire [38].

### 2.3. Episodic Memory Paradigm

For the encoding phase of each session, 40 different mono- and disyllabic concrete nouns were selected from 10 different categories, 4 words each [39, 40]. As categories did not overlap between sessions, a total of 120 words were used during encoding. Across study days, words were balanced regarding length, valence, arousal, and frequency. For each of the MR sessions, an additional 40 words (80 in total, none of them used in the encoding phase in any of the sessions), half of which was drawn from the same category as the encoded words (similar) while the other half was unrelated to previously presented words (dissimilar), were randomly intermingled in the recognition task. The number of words was chosen in accordance with a previous behavioural study [18] wherein no ceiling effects emerged.

The verbal episodic memory task was computerized and programmed in E-Prime 3.0 software (Psychology Software Tools, Pittsburgh, PA; Figure 1(b)). For encoding, words were presented in a randomized order on a screen in white on a black background in three successive blocks. Each word only occurred once per block. After a priming fixation cross, each word was displayed for 1 s. The duration of the interstimulus interval, during which a fixation cross remained on the screen, was jittered between 1 and 6 s (mean = 3 s). Participants were instructed to memorize the presented words to the best of their ability. After each block, participants were prompted to immediately recall the remembered words in 2 min. The responses were logged in separate audio files. The retention phase lasted for ~30 min, during which the post tDCS MR spectra were acquired and the tDCS dismounted outside of the scanner. During the retrieval phase, participants performed a free delayed recall. Performance in the free delayed recall was evaluated in terms of remembered words. The timing of the recalled words, however, was not recorded. During the MR sessions, participants also completed a recognition task outside of the scanner. Therein, they were asked to indicate via a button press whether the presented word was previously shown during the encoding phase or not (i.e., was a distractor). Recognition performance was evaluated in terms of the sensitivity index *d*′ and median reaction times.

### 2.4. tDCS

During the two sessions in the MRI scanner, participants received either sham or anodal tDCS in a randomized, counter-balanced order. To ensure the blinding of the experimenter and the study participant, the DC-Stimulator MR (neuroCare Group GmbH, Ilmenau, Germany) was set to “study mode,” allowing its operation with blinded codes (allotted by a statistician at the CTU Bern) for the two conditions. It delivered a 1 mA current to the brain using a pair of rubber electrodes (5 cm × 7 cm), which were covered with EEG gel. The anode was centered over the F3 position corresponding to the 10-20-EEG system of the electrode placement [41] to target the left DLPFC (Figure 1(c)). The cathode was placed on the contralateral supraorbital area (above the right eyebrow). For real anodal tDCS, the current was ramped up for 15 s, kept constant at 1 mA for 20 min, and ramped down for 15 s afterwards. In the sham condition, using the same ramping up and down procedure as in the active one, the current was only kept constant at 1 mA for 30 s. This procedure elicits the same itching sensation occurring at the beginning of the longer-lasting stimulation, thus ensuring the best possible blinding of the participant [42]. Stimulation started 5 min prior to the first encoding block to permit a build-up of tDCS effects on cortical excitability as suggested by previous research [43].

### 2.5. MRI/MRS Data Acquisition

MR data were acquired on a 3T Siemens MR scanner (MAGNETOM Prisma, Siemens, Erlangen, Germany) using a 20-channel head coil. After recording scout images, high-resolution anatomical T1-weighted images were acquired with a magnetization-prepared acquisition gradient echo (MPRAGE) sequence (160 slices, 256 × 256 mm matrix, 1.0 mm isotropic resolution, TR = 2300 ms, TE = 2.98 ms, flip angle = 9°, and GRAPPA factor = 2). MRS data were acquired using sequences derived from the svs_se sequence (MEGA-PRESS sequence (work in progress package, WIP 859G), Siemens; TR = 1500 ms, TE = 68 ms, 20 × 20 × 20 mm voxel, 208 averages, and TA = 10 min30 s). The voxel was manually positioned in the left DLPFC, covering the middle frontal gyrus (Figure 1(d)). The outer lateral side was aligned to the curvature of the head, to contain as much grey matter as possible while excluding the meninges. MRS data were acquired before and after (pre and post, respectively) the stimulation period, which coincided with the encoding phase of the cognitive task. The presence of the electrodes had no noticeable negative impact on shimming or other artefacts.

### 2.6. MRS Analysis

Analysis was performed with the jMRUI software package [44], using the spectrIm plugin [45].

After water removal, the MRS signal was smoothed with a 3 Hz Lorentzian filter and offset corrected in the frequency domain. Spectra were quantified using the TDFDFit algorithm [46], which is an iterative nonlinear least-squares fitting algorithm. Two TDFDFit quantification models were defined (assuming Voigt line shapes) to quantify the nonedited spectrum and the edited difference spectrum.

The nonedited model (TE = 68 msec) contained a basis set including, apart from a model for residual water, choline, creatine, glutamate, glutamine, N-acetylaspartate (NAA), and lactate. Maximal prior knowledge was imposed in order to minimize the Cramér-Rao minimum variance bounds. The latter means that for NAA, all 5 parameters (i.e., peak area, frequency shift, Lorentzian width constant, zero order phase, and Gaussian width constant) were fitted, while for the remaining metabolites, the relative frequency shifts and Lorentzian/Gaussian line width parameters were assumed to be fixed relative to NAA. From this fit, the total creatine (tCr) signal was used for further quantitative analysis. The spectra were fitted in the 1.0–5.1 ppm offset range. The edited difference spectrum was fitted using a model that consisted of a basis set containing experimental patterns for GABA, glutamate, and glutamine, which were obtained by measuring phantom solutions of the metabolites. Prior knowledge was applied such that for the glutamate pattern, all 5 parameters were fitted; for glutamine, only the peak area parameter was fitted; and for GABA, the frequency shift was fixed towards glutamate. In order to account for the residual creatine signal present at 3 ppm, the Cr-CH3 singlet was also part of the model. GABA and Glx values (i.e., the combined measure of glutamate and glutamine) were obtained from this edited difference spectrum type. The offset range 1.9–5.1 ppm was selectively fitted (Figure 1(e)).

The fit quality number (FQN) was determined [46, 47] as the ratio between the sum of least squares and the variance of the noise of the signal. Therein FQN = 1.0 indicates an ideal fit, FQN < 0 signifies overfitting, and FQN > 1.0 indicates a nonideal fit. Data with a poor spectral fit quality (if the FQN > 2.0 for nonedited spectra and FQN > 1.5 for difference spectra) were excluded (young: *n* = 7, elderly: *n* = 5; S1). Due to coediting, the GABA signal incorporates the macromolecular baseline; wherefore, it is henceforth referred to as GABA+.

The proportions of grey matter (GM), white matter (WM), and cerebrospinal fluid (CSF) in the voxel were quantified by means of an in-house written automated segmentation tool, based on the pixel values of the T1-weighted MPRAGE scans. Following the recommendation by Harris and colleagues [48], GABA+ peaks were corrected for GM and WM, with the assumption that the concentration of GABA in WM is half the amount of the concentration of GABA in GM. Peaks of glutamatergic metabolites were corrected for the proportion of GM in the voxel, whereas tCr peaks were corrected for the total amount of brain tissue within the voxel. GABA+ and glutamatergic values are further reported as ratios of tCr, i.e., GABA+ : tCr and Glx : tCr.

### 2.7. Statistical Analysis

Data sets for one young and one elderly participant, who discontinued their participation in the study, were henceforth excluded from further analysis. Further, technical problems with the presentation of words and recording of responses, as well as a premature interruption of MRS acquisition, led to incomplete behavioural (young: *n* = 6, elderly: *n* = 5) and MRS data sets (young: *n* = 4, elderly: *n* = 1), which were excluded from the respective subanalyses (S1).

We conducted separate analyses for young and elderly populations due to expected different effect sizes and diverging performance levels. However, to increase the power of our tests, we exploratively pooled all participants in additional analyses including age group (young vs. elderly) as a categorical control variable. Following a reviewer's suggestion, we tested the contribution of gender to stimulation effects by including this variable as a covariate in the respective ANOVAs. As this did not alter any of the results, we only report outcomes from the simpler model. All statistical analyses were carried out in MATLAB (version R2018a, MathWorks, Inc., Natick, Massachusetts, USA), except moderation analyses, which were performed in SPSS (version 25.0, IBM Corp., Armonk, NY, USA). *Q*‐*Q* plots were inspected to check for normality (Figure S2), and *α*-levels of *p* < 0.05 denote statistical significance.

#### 2.7.1. Episodic Memory Paradigm

To test for overall stimulation effects in immediate free recall, we used 2 × 3 repeated measure ANOVAs on the number of remembered words with stimulation (real vs. sham) and retrieval (immediate retrievals 1–3) as within-subject factors. Further, we tested for tDCS-induced cognitive benefits in delayed recalls, using 2 × 2 repeated measure ANOVAs on the number of remembered words with stimulation (real vs. sham) and retrieval (delayed recalls 1–2) as within-subject factors.

Based on the results in our previous study [18], we performed moderation analyses to test whether the baseline performance in the episodic memory task affects the tDCS-related gains in free verbal recall during the MR sessions. Owing to the switch to a crossover design as opposed to the previously employed parallel study design, here, the baseline assessment of verbal memory performance is completely unaffected by stimulation at first exposure to the task. Additionally, the altered design also allowed us to use corresponding recalls across study days, focussing on performance in delayed recall 1. For the moderation analyses, we applied the SPSS MEMORE macro (version 2.0) [49] with stimulation (real vs. sham) as the focal predictor, remembered words in delayed recall 1 at baseline as the moderator variable, and remembered words in delayed recall 1 during the MR sessions as the outcome variables.

To test for training effects on immediate recalls, we performed separate 3 × 3 repeated measure ANOVAs on the number of remembered words with condition (baseline, sham, and real) and retrievals (immediate retrievals 1–3) as within-subject factors. Similarly, we tested for training effects on delayed recall 1 by way of a one-way ANOVA on three groups (baseline, sham, and real).

Data from the recognition task was tested for group differences in terms of the sensitivity index *d*′ as well as median reaction times of correct responses by means of Wilcoxon signed rank tests. Additional exploratory Wilcoxon signed rank tests were conducted on reaction times of correct responses for similar and dissimilar distractor words.

#### 2.7.2. Neurotransmitter Measures

To compare changes in MRS peak areas, GABA+ : tCr, Glx : tCr, and E/I ratios (calculated as the ratio of Glx : tCr to GABA+ : tCr) were subjected to 2 × 2 repeated measure ANOVAs with stimulation (real vs. sham) and time (pre vs. post) as between-subject factors, for young and elderly participants.

Additional *t*-tests were performed on the percent of change of GABA+ : tCr and Glx : tCr levels as well as for E/I ratios depending on the stimulation condition.

#### 2.7.3. Relationship between Neurotransmitter Levels and Memory Performance

To explore the relationship between episodic memory performance and neurometabolites, we conducted correlation analyses between levels of GABA+ : tCr, Glx : tCR, and E/I ratios before tDCS as well as their respective percentage of change and words recalled in delayed recall 1 separately for each stimulation condition as well as pooled across conditions. The Bonferroni-Holm method [50] was applied to correct for 18 multiple comparisons within each age group as well as for data pooled across populations.

Further, we tested the predictive value of neurotransmitter levels measured prior to the stimulation on subsequent tDCS-related cognitive gains, applying the SPSS PROCESS macro (Version 2.16, [51]). Separate moderation analyses were conducted with GABA+ : tCr, Glx : tCR, and E/I ratios as moderator variables. In each of these analyses, stimulation (real vs. sham) was entered as the focal predictor and remembered words in delayed recall 1 as the outcome variable.

## 3. Results

Both young and elderly participants as well as the experimenter were effectively blinded to the stimulation condition (all *p* ≥ 0.07). The strength of side effects (for overview, see S3) did not differ significantly between stimulation conditions (all *p* ≥ 0.06) except for scalp pain (*p* = 0.003). However, reporting scalp pain was not associated with the correct identification of the stimulation condition (*p* = 0.42).

### 3.1. Episodic Memory Paradigm

**Figure 2:**
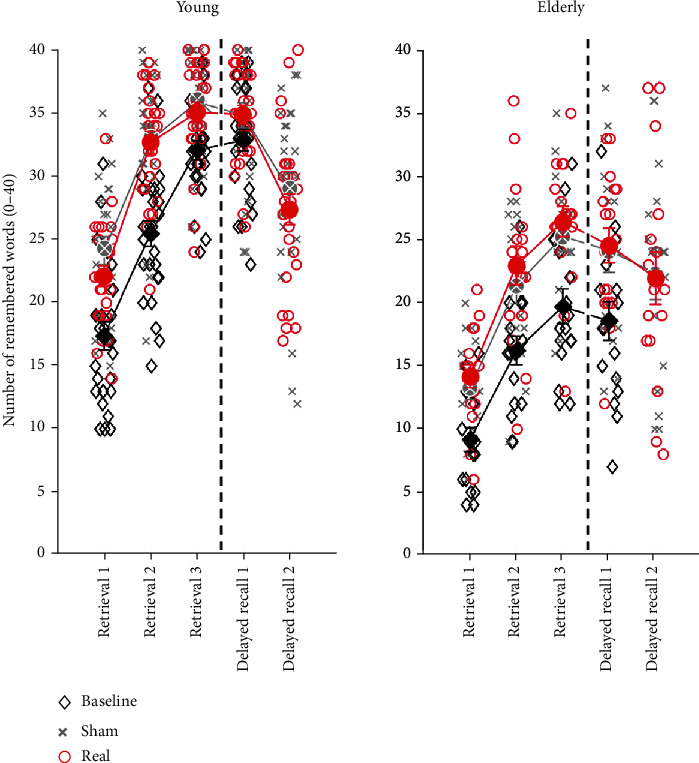
Performance in verbal episodic memory task. Number of words recalled during encoding and retrieval phase according to session (black diamonds: baseline; grey crosses: sham stimulation; red circles: real anodal tDCS) in young and elderly participants. Note that the delayed recall 2 was only conducted after MR sessions and not for the baseline session. Large symbols: mean ± SE; small symbols: performance of single subjects.

Neither young (*F*_(1, 25)_ = 1.26, *p* = 0.27) nor elderly (*F*_(1, 16)_ = 2.38, *p* = 0.14) participants showed a main effect of stimulation across the 3 immediate retrievals but only a significant main effect of retrieval round (young: *F*_(2, 24)_ = 218.99, *p* < 0.001, elderly: *F*_(2, 15)_ = 122.95, *p* < 0.001, Figure 2). Likewise, no stimulation effect emerged for either the young (*F*_(1, 25)_ = 0.47, *p* = 0.50) or elderly (*F*_(1, 16)_ = 0.001, *p* = 0.97) on the two delayed recalls. Additionally, the young (*F*_(1, 25)_ = 62.4, *p* < 0.001) but not elderly (*F*_(1, 16)_ = 3.20, *p* = 0.09) showed a significant main effect on the retrieval round, with a better performance in delayed recall 1.

Including baseline performance in delayed recall 1 as a moderator of tDCS-induced performance gain during the MR sessions produced no significant transitioning point in either of the two study populations. The additional 3 × 3 repeated measure ANOVAs on immediate recalls across all three study sessions revealed a significant main effect of condition in young (*F*_(2, 50)_ = 27.86, *p* < 0.001) and elderly (*F*_(2, 32)_ = 34.28, *p* < 0.001) participants that was driven by the improved performance in the MR sessions as compared to the baseline assessment.

Irrespective of stimulation condition, ceiling effects emerged with respect to percentages of correct responses in young (98.77 ± 1.84% [mean ± SD]) and elderly (95.44 ± 3.69% [mean ± SD]) participants in the recognition task. Further tests revealed no significant differences between stimulation conditions in sensitivity index *d*′ (young: *Z* = 0.50, *p* = 0.62, elderly: *Z* = 1.02, *p* = 0.31) or reaction times for correct responses (young: *Z* = −0.84, *p* = 0.40; elderly: *Z* = 1.16, *p* = 0.25). In young participants, additional exploratory analyses of reaction times showed no significant differences in stimulation conditions for similar (*Z* = −1.01, *p* = 0.31) and dissimilar (*Z* = −0.53, *p* = 0.60) distractor words. The elderly showed a tendency for shorter reaction times in the real tDCS condition for similar distractor words (*Z* = 1.96, *p* = 0.05) while no such difference emerged for dissimilar distractors (*Z* = 1.52, *p* = 0.13).

Exploratory repeated measure ANOVAs with the additional between-subject factor age group showed a significant interaction between retrievals and age group for delayed recalls (*F*_(1, 41)_ = 9.26, *p* = 0.004) but not for immediate retrievals (*F*_(2, 82)_ = 0.92, *p* = 0.40). Post hoc comparisons confirmed the consistently higher performance in young compared to elderly participants with a mean difference of 9.90 words, *p* < 0.001, in immediate retrievals and 8.76 words, *p* < 0.001, in delayed recalls. The three-way interaction between stimulation condition, retrievals, and age group revealed no significant effect for either immediate retrievals (*F*_(2, 82)_ = 0.28, *p* = 0.75) or delayed recalls (*F*_(1, 41)_ = 0.05, *p* = 0.83).

### 3.2. Neurotransmitter Measures

There were no significant differences in fit quality numbers and grey or white matter content between the stimulation conditions in either of the two age groups (all *p* ≥ 0.24).

**Figure 3:**
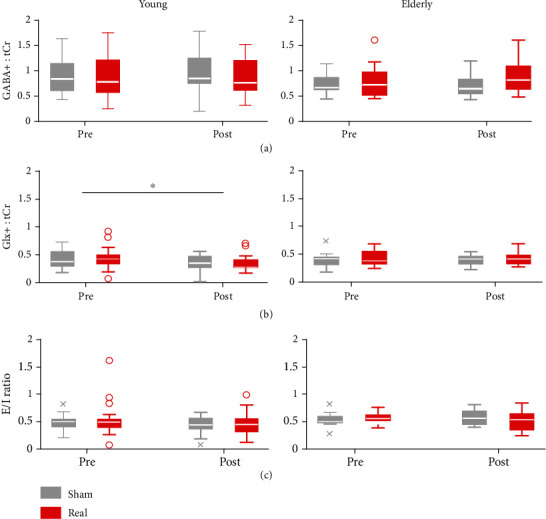
Neurometabolite levels pre and post anodal tDCS in young and elderly participants. (a) In both populations, no significant interactions between time and stimulation emerged for GABA+:tCr. (b) Young participants showed a reduction of Glx:tCr levels over time irrespective of stimulation condition. This effect was absent in elderly. (c) E/I ratios showed no significant interaction between time and stimulation conditions in either of the two populations.

2 × 2 repeated measure ANOVAs in young participants showed no significant interaction between stimulation and time in GABA+ : tCr (*F*_(1, 17)_ = 2.35, *p* = 0.14, Figure 3(a)) or Glx : tCr (*F*_(1, 18)_ = 0.04, *p* = 0.84, Figure 3(b)) levels. For Glx : tCr levels, only the main factor time reached significance (*F*_(1, 18)_ = 8.07, *p* = 0.01). Likewise, no significant interaction arose for the E/I ratio (*F*_(1, 18)_ = 0.20, *p* = 0.66, Figure 3(c)). In young participants, *t*-tests on the percent of change of neurometabolites revealed no significant differences between stimulation conditions in GABA+ : tCR (*t*_(17)_ = 1.56, *p* = 0.14), Glx : tCr (*t*_(18)_ = −0.97, *p* = 0.34), or E/I ratios (*t*_(15)_ = −0.96, *p* = 0.35).

In elderly participants, no significant interaction between stimulation and time manifested for GABA+ : tCr (*F*_(1, 12)_ = 2.43, *p* = 0.14, Figure 3(a)) or Glx : tCr (*F*_(1, 12)_ = 0.10, *p* = 0.76, Figure 3(b)) levels. For GABA+ : tCr levels, the main factors stimulation (*F*_(1, 12)_ = 4.98, *p* = 0.05) and time (*F*_(1, 12)_ = 4.92, *p* = 0.05) reached marginal significance. For the E/I ratio, the interaction term became marginally significant (*F*_(1, 12)_ = 4.68, *p* = 0.05, Figure 3(c)). Furthermore, the stimulation condition did not influence the percentage of change in GABA+ : tCr (*t*_(12)_ = −1.92, *p* = 0.08) or Glx : tCr (*t*_(12)_ = 0.26, *p* = 0.80) over time. However, there was a significant difference in percentage of change for the E/I ratio (*t*_(12)_ = 2.50, *p* = 0.03) with real anodal tDCS inducing a greater reduction of cortical excitability.

Pooling MRS data from young and elderly participants in an additional ANOVA with age group as a between-subjects, no significant interaction between stimulation and time emerged for GABA+ : tCr, Glx : tCr, or E/I ratios. Further, there was no significant main effect of the age group in any of the neurometabolite measures.

### 3.3. Correlations between Episodic Memory and Neurotransmitter Measures

No significant relationships were observed between delayed recall performance and baseline GABA+ : tCr, Glx : tCr, or E/I ratios nor were there respective changes for either the young or elderly participants. The same holds true for data pooled across age groups.

**Figure 4:**
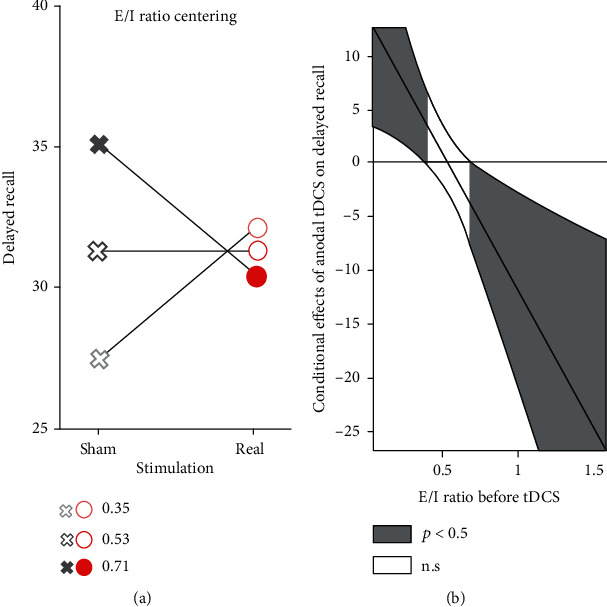
Differential benefits of anodal tDCS. (a) Moderation effect of E/I ratio prior to stimulation on the relationship between stimulation and remembered words in delayed recall 1 for three centerings of E/I ratio (−SD, mean, and +SD). Conditional effects of performance in delayed recall 1. (b) Johnson-Neyman confidence bands indicate ranges of E/I ratios in which the latter has a significant predictive value.

When conducted separately for each of the two age groups, none of the moderation analyses resulted in a significant model. Likewise, when pooling the data of young and elderly participants, models with GABA+ : tCr and Glx : tCr measures alone did not reach significance. However, a predictive value of the E/I ratio prior to the application of tDCS was indicated by the overall significant fit of this model (*F*_(4, 67)_ = 23.90, *p* < 0.001, *R*^2^ = 0.59). More precisely, low initial E/I ratios led to a higher number of remembered words in the delayed recall 1 under real stimulation as compared to the sham while high initial E/I ratios favoured the sham over real tDCS (Figure 4(a)). In this model, the simple effects of stimulation (*t*_(67)_ = 2.73, *p* = 0.008) and E/I ratio (*t*_(67)_ = 2.52, *p* = 0.014), and age group as a covariate (*t*_(67)_ = −9.41, *p* < 0.001) as well as their interaction (*t*_(67)_ = −2.78, *p* = 0.007) were significant. Including the latter in the model led to a significant increase in its explained variance (*R*^2^_change_ = 0.05, *F*_(1, 67)_ = 7.71, *p* = 0.007). Johnson-Neyman confidence bands revealed that this interaction was significant for the 16.7% of the lowest initial E/I ratios and the 9.7% highest ones (Figure 4(b)).

## 4. Discussion

This study builds on our previous research in which we demonstrated that tDCS effects are differential, favouring the initial low performers in a verbal episodic memory task [18]. While only a relative homogenous population of healthy young adults with an age-appropriate cognitive performance participated in the latter, we aimed to increase the clinical validity of this finding by including elderly participants, whose episodic memory capacity is known to be decreased as a function of old age [52–54]. At the same time, greater effect sizes of stimulation emerged in healthy and pathological ageing [20, 55]. Despite this promising outlook, here, we could not reproduce the previous results in young adults. Similarly, the beneficial impact of tDCS in the elderly was restricted to the more demanding subcondition in a recognition task. Furthermore, we augmented our previous study with acquisitions of MR spectra, focussing on the assessment of Glx and GABA levels before and after the application of anodal tDCS. While the stimulation did not significantly influence Glx or GABA in either of the two populations, their ratio, denoting cortical excitability, decreased after tDCS in the elderly.

A prior sample size calculation based on our previous study in healthy young adults (Cohen′s *d* = 0.61; [18]) indicated that we would find a robust effect with 30 participants. The required sample size in healthy elderly was based on the observed effect size (Cohen′s *d* = 1.01) in a similar study by Sandrini and colleagues [11], suggesting a required sample size of 20 participants. Given the appropriate sample sizes, the absence of a behavioural tDCS effect in this study may be attributed to the change in study design from parallel to crossover, which we chose to reduce interindividual variability. Across immediate retrievals 1 to 3 and delayed recall 1 at baseline, young participants recalled a comparable number of words as in our previous study [18]. However, during the following study days, the familiarity with the task led to a significantly improved free recall regardless of stimulation condition. While contributing to an efficient blinding of participants, sham tDCS is not supposed to induce cognitive benefits [42]. Hence, the difference in performance between baseline and sham condition should be attributed to participants being more accustomed to the task. The associated ceiling effects potentially precluded the emergence of tDCS-induced heightened recall scores in young participants. As opposed to logical memory tests, list learning tests do not generate as pronounced practice effects [56], even less so when parallel versions are applied [57]; we did not anticipate this effect. While we ensured the equivalence of the employed word lists across study sessions by controlling for length, valence, arousal, and frequency of the items, we could not prevent the use of more elaborate mnemonic strategies upon familiarization with the task. Different approaches to the word list learning task on separate study days are not only problematic in consequence of the reduced number of low performers for whom an effect of stimulation is hypothesised. Moreover, those different approaches also involve a different pattern of activation across brain regions other than the ones identified to be involved in genuine episodic memory processes. In the case of verbal episodic memory, the left DLPFC is crucially involved in the encoding of memory contents [2, 3]; wherefore, we chose this cortical region as a target for concurrent anodal tDCS during task performance. The consideration of the target site is even more crucial in consideration of elderly participants as their brain activation patterns change in response to task demands [58], markedly described by the model of hemispheric asymmetry reduction in older adults (HAROLD; [59]). This raises the question of whether tDCS should be adapted to support the more bilateral activity pattern naturally occurring in the brains of the elderly or rather to reverse the changes that are associated with ageing by resetting the cortical activity pattern to the state found in younger individuals. As two studies demonstrated that the restoration of a more youth-like activity pattern via anodal tDCS in the elderly correlated with an improved performance in cognitive task performance [60, 61], we opted against a switch of target region across populations and used the same setup for unilateral anodal tDCS in both the young and elderly.

Here, the elderly experienced only marginal stimulation gains in the recognition task, showing reduced reaction times when correctly identifying similar distractors, which is the more difficult task compared to the correct identification of dissimilar distractors. This agrees with the prior hypothesis of tDCS gains being restricted to higher task demands [17]. Nevertheless, given the absence of more substantial stimulation benefits (i.e., in free recalls) in the older population, future studies might explore whether the effectivity of the stimulation improves when adjusting the target to the compensatory requirements of older brains. Apart from spatial considerations regarding the target, attention needs to be paid to the temporal alignment of tDCS to the task-related brain activity. In principle, a concurrent application of the stimulation with the task execution, as compared to applying the stimulation prior to the task, has been proven most effective in young and especially in elderly adults [62, 63]. Given that the encoding phase during which tDCS was applied in this study consisted of encoding proper and immediate recalls, the task-related brain activation pattern did not consistently overlap with the target region of tDCS. This might have attenuated possible stimulation effects. However, the placement of the cathode over the right supraorbital region, which is not involved in memory processing, should have prevented a detrimental impact of tDCS on the activation of the right DLPFC during immediate retrievals. Aside from influencing encoding and retrieval phases, even longer-lasting cognitive benefits arose upon the application of tDCS during the consolidation of memory contents [64]. Additionally, the use of priming protocols, which rely on lowering the threshold for plasticity induction, is conceivable to strengthen the tDCS effects as previously demonstrated in the motor cortex [65].

For the most part, earlier studies focussed on the separate exploration of tDCS effects on behaviour on the one hand and the influence of the stimulation on neurophysiological measures on the other hand. Only the combination of all three aspects (i.e., tDCS, behavioural, and neurophysiological measures) in a single study provides an adequate setting to test if and how changes induced by tDCS on a neurophysiological level produce performance changes in cognitive paradigms [66]. Heretofore, research in the primary motor cortex is the vanguard in this kind of combinatorial study design and produced relatively consistent outcomes. However, MR spectra of glutamatergic metabolites and GABA, acquired following stimulation of brain regions other than the primary motor cortex, yielded less uniform results. Notably, anodal tDCS to the left DLPFC only led to increased levels of glutamatergic metabolites in the striatum while no changes in Glx or GABA levels were detected in the target region [67]. Although tDCS effects on neurotransmitters also occur without simultaneous engagement in a task, greater focality of the stimulation and thus greater effect sizes are expected in the case of the interaction of tDCS with task-related physiological brain activity [68], which we aimed for in the current study. Nevertheless, we did not find the hypothesised reduction of GABA or elevation of the Glx level in the stimulated left DLPFC. This lack of neurotransmitter changes is by no means attributable to a less critical role of Glx and GABA in the function of this brain region. On the contrary, by way of its involvement in the induction of long-term potentiation (LTP) as the basis of learning and memory [69], Glx mediates cognitive processes. Along these lines, prefrontal Glx levels predicted the reversal learning task performance in marmosets [70] while reduced glutamate levels correlated with cognitive impairment in old age [71]. Similarly, GABA as the main inhibitory neurotransmitter of the brain shapes cortical activation [72, 73]. Increased GABA levels are associated with greater difficulties of healthy participants when facing a higher working memory load [74] but also with episodic memory dysfunction in diabetes type 2 patients [75]. Interference with GABA transmission on the other hand ameliorated age-related cognitive deficits in rats [24] while the reduction of the GABA level via anodal tDCS reinstated memories in human subjects [76]. In the absence of consistent tDCS effects on either of the two neurotransmitter levels on their own, it has been suggested that the regional cortical excitation/inhibition (E/I) balance may be a more meaningful measure to evaluate network activity efficiency as Glx and GABA contribute in a complementary fashion [32]. Indeed, here, the elderly showed a greater reduction of the E/I ratio following anodal tDCS as compared to sham stimulation. Even though contrary to expectations, this result confirms the greater meaningfulness of this combined measure of cortical excitability.

Independent of the specific effect of the stimulation, the responsiveness of the system to tDCS depends on how efficiently it performs without the stimulation. If it already performs at its homeostatic optimum, characterized by a finely tuned E/I balance, additional input as provided by tDCS may be resisted by the system to remain within the homeostatic limits to prevent overexcitation [77], which may have resulted in the observed effect. The decrease in Glx over time that we observed independent of stimulation condition in young participants points in the same direction. While the brains of healthy young adults can be expected to function at their age-appropriate optimum and, hence, do not possess the room for improvement as a prerequisite for tDCS-induced gains, the same principle may hold true for elderly individuals with a high cognitive reserve [78]. While we hypothesised greater effect sizes in the elderly, the high educational level in the study population, with only two participants reporting less than 12 years of education, may also have prevented further cognitive improvements in the older population. Whether greater effect sizes arise in the elderly with lower educational levels remains to be tested. Apart from neurometabolite changes following stimulation, Filmer and colleagues demonstrated that a higher inhibitory tone at baseline, defined as a higher GABA/Glx ratio, entailed a greater disruptive effect of cathodal tDCS to response selection training gains [79]. At the same time, another study yielded no evidence that cortical excitability at baseline could be adduced to predict the effects of anodal or cathodal tDCS on working memory performance [80]. While the latter is in line with the main findings of our study, our exploratory analysis acknowledged a predictive value of the E/I ratio on cognitive tDCS gains insofar as a lower excitability at baseline is connected to a better performance in the delayed recall. Conversely, higher excitability at baseline indicated a less favourable impact of the stimulation. This finding further endorses the notion that tDCS is more beneficial in systems, which operate on suboptimal levels, e.g., regarding excitability.

In young women, the response to tDCS may also depend on their menstrual cycle and the respective hormonal status. In this, high levels of estrogen are associated with not only elevated structural [81] but also functional plasticity [82, 83]. How this factor influenced the outcome of this study cannot be conclusively determined since sessions were not clocked according to the participants' cycle. However, regarding the intended application of tDCS as a treatment for cognitive decline in old age, this issue is only of minor importance.

Generally, the impact of tDCS is limited due to a low tissue penetration depth of the applied current [84, 85], a problem which is further aggravated in the elderly who, tendentially, exhibit an increased skull thickness [86]. On the other hand, ageing is accompanied by a higher porosity of osseous structures [87], which may amplify the permeability for tDCS. Nevertheless, decreases in cortical thickness with age [88] in combination with local atrophies [89, 90] lead to a greater shunting of the current. Considering those impediments, several improvements to the current experimental procedures are conceivable to test and exploit the full potential of tDCS. Concerning the former, employing a high-definition electrode setup, which ensures a more focal stimulation of target brain areas [91] might be advantageous to exclude unforeseen interactions between the applied current and brain regions other than the target one. This, however, hinges on the reliable identification of a single target region or multiple target regions, which, in the case of cognitive tasks in particular, is in itself a complicated venture because cortical activity patterns during the execution of different tasks overlap [92]. As discussed above, the choice of a target region is even more complex in the elderly due to compensational activity patterns [59]. Demonstrably, electric field distributions strongly overlap in young and older adults [93]. However, to ensure that the applied electric current arrives at the identified target region, older participants may require different electrode arrangements to compensate for altered current density distributions owing to brain atrophy accumulated during ageing [89, 94]. Even more promising than adjustments of electrodes might be the repeated application of tDCS within a single session [95] or over several days [96]. Apart from enhancing the immediate effect of the stimulation due to the consolidation of changes in neuroplasticity, this procedure is also known to produce longer-lasting cognitive gains up to several months [97], which in turn increases the value of the treatment in a clinical setting.

Provided that tDCS has the hypothesised impact on neurometabolites, the failure to reliably detect those might also be related to the current limitations of MRS. In this regard, MRS measurements cannot distinguish between neurometabolites from different functional pools with their respective tonic or phasic dynamics [98]. Whether and how they are differentially influenced by tDCS cannot be resolved by the study at hand and would need to be the subject of future studies. Moreover, coediting leads to overlapping spectral peaks of Glu and Gln as well as GABA with the macromolecular baseline, thus further reducing the explanatory power of the measures as to the underlying physiological processes [99]. Some of these current drawbacks could be redeemed with the use of advanced MRS techniques. For instance, it is to be expected that the semi-LASER-based [100–102] MEGA-edited MRS [103] will increase the sensitivity of both Glx and GABA detection due to its increased RF-pulse bandwidth and spatial selectivity, which leads to a less pronounced chemical shift displacement-related signal cancelation over the volume of interest. Furthermore, the use of 7T-MRS is promising as it allows not only the disentanglement of glutamate and glutamine peaks in the spectrum [104] but also the subtraction of the macromolecular baseline from the GABA signal [105]. The latter is of particular interest since suppressing the macromolecular spectral components increased the correlation of GABA levels with individual discrimination thresholds for vibrotactile and visual stimuli [106] and may thus contribute to a better understanding of the relationship between neurometabolites and behaviour. Further, functional MRS as opposed to the current resting state assessment of MRS spectra could help to identify more immediate effects of the stimulation, which may be obscured by the long acquisition times over several minutes [107]. Comprehensive research in these directions may ultimately lead to the development of a combined tDCS-MRS protocol specifically adapted to prefrontal areas following the example set by the one proposed for the primary motor cortex [108].

## 5. Conclusion

In accordance with an accumulating number of studies reporting null effects for tDCS on the group level, our study could not conclusively determine the effects of anodal tDCS on episodic verbal memory performance or on neurotransmitter levels in the left DLPFC. Nonetheless, our exploratory findings demonstrate that the importance of evaluating neurophysiological along with behavioural measures in sufficiently powered future tDCS studies persists. Both understanding the neurophysiological underpinnings of the tDCS-induced cognitive benefits evidenced in multiple other studies and the identification of biomarkers to predict stimulation outcomes are indispensable to refine the potential of tDCS as a valuable cognitive therapy option in healthy and pathological ageing. As opposed to using tDCS merely in a standard manner, a higher potency is ascribed to an individualized treatment that takes distinct patient characteristics into account and relies even more on an exhaustive knowledge as to the mechanism of action of the stimulation method.

## Figures and Tables

**Table 1:** Demographic characteristics of young and elderly participants (mean ± SD).

	Young (*n* = 33)	Elderly (*n* = 22)	
Age (years)	24.5 ± 2.6	67.3 ± 4.4	*p* < 0.001
Gender	20 females, 13 males	11 females, 11 males	*p* = 0.58
Education (years)	16.8 ± 2.1	15.2 ± 3.8	*p* = 0.05
WST	32.7 ± 3.4	33.4 ± 3.7	*p* = 0.49
EHI	94.7 ± 9.2	95.1 ± 11.6	*p* = 0.89
BDI	3.0 ± 3.1	5.0 ± 3.3	*p* = 0.02
MoCA	—	26.2 ± 2.2	

## Data Availability

Data will be available upon request to the corresponding author.
