# Supplementary material for: No Effect of Anodal tDCS on Verbal Episodic Memory Performance and Neurotransmitter Levels in Young and Elderly Participants
Source: Neural Plast. 2020 Sep 22;2020:8896791. doi: 10.1155/2020/8896791 (PMC7528151; doi:10.1155/2020/8896791)
Supplement: Supplementary Materials [file 8896791.f1.docx]

**Supplements**

S1


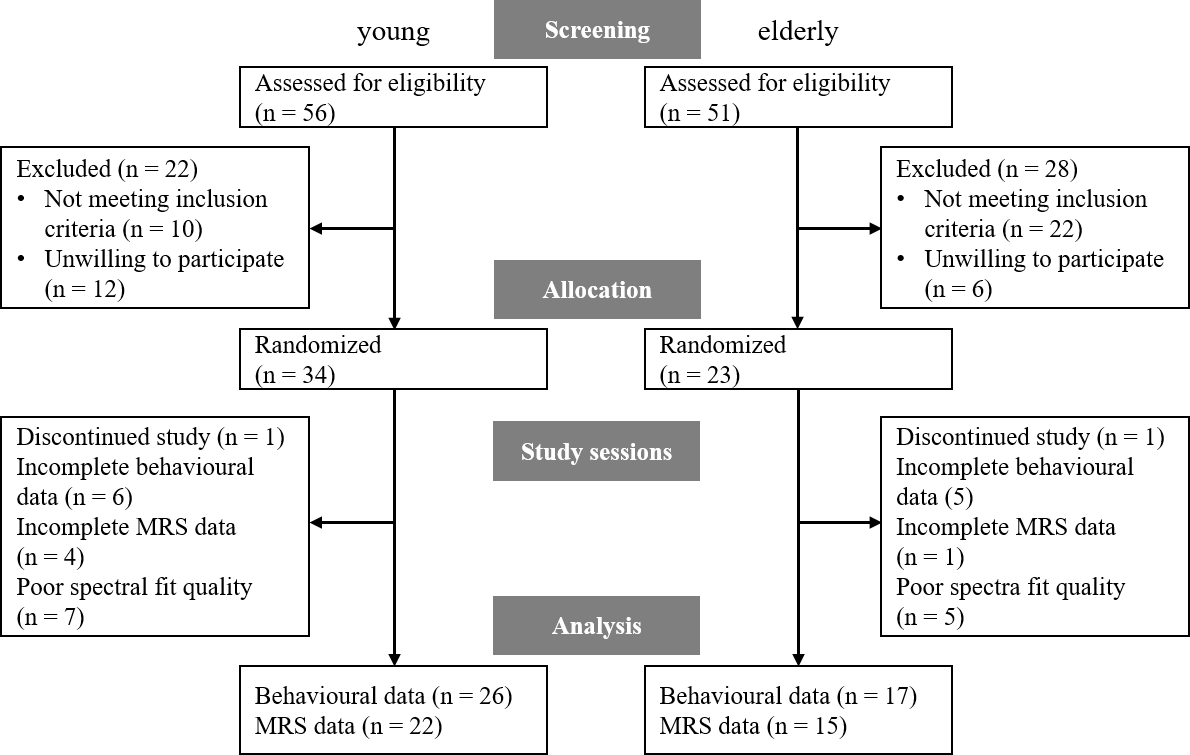


CONSORT flow diagram.

S2


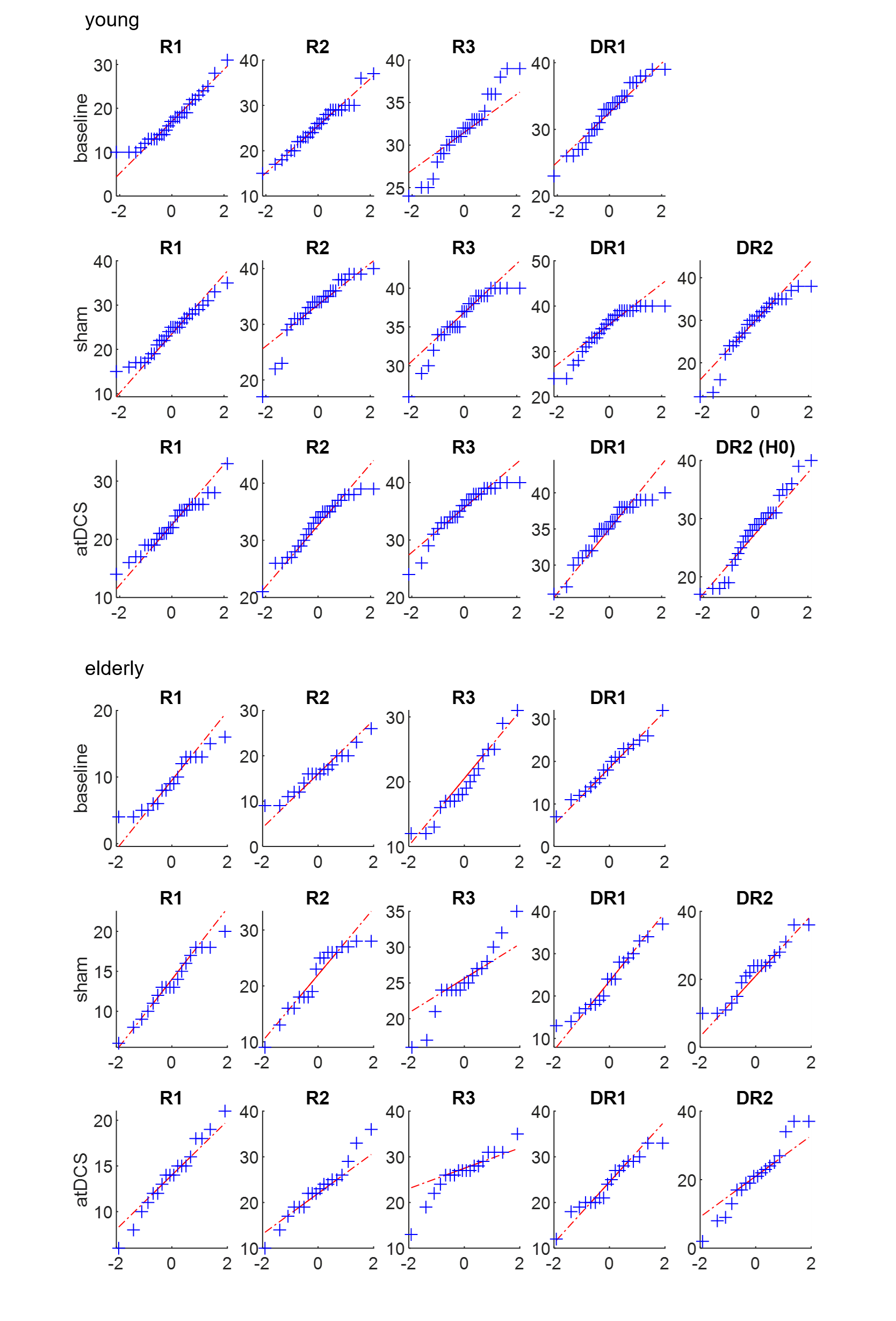


Q-Q plots of free recall performances in young and elderly participants split according to session. Despite ceiling effects in later recalls (R3 and DR1 in particular) in young participants, no considerable deviation from normal distribution.

S3

| Side effects | young (n = 33) | | elderly (n = 22) | | all (n = 55) | |
| --- | --- | --- | --- | --- | --- | --- |
|  | sham | real tDCS | sham | real tDCS | sham | real tDCS |
| Headache | 1.38 ± 0.66 | 1.40 ± 0.80 | 1.19 ± 0.51 | 1.10 ± 0.30 | 1.30 ± 0.61 | 1.28 ± 0.66 |
| Neck pain | 1.22 ± 0.66 | 1.28 ± 0.63 | 1.10 ± 0.30 | 1.05 ± 0.22 | 1.17 ± 0.55 | 1.19 ± 0.52 |
| Scalp pain | 1.09 ± 0.30 | 1.59 ± 0.95 | 1.05 ± 0.22 | 1.14 ± 0.48 | 1.08 ± 0.27 | 1.42 ± 0.82 |
| Tingling | 2.13 ± 0.94 | 2.15 ± 0.85 | 1.48 ± 0.60 | 1.43 ± 0.75 | 1.87 ± 0.88 | 1.87 ± 0.88 |
| Itching | 1.47 ± 0.92 | 1.88 ± 1.16 | 1.24 ± 0.77 | 1.33 ± 0.58 | 1.38 ± 0.86 | 1.66 ± 1.00 |
| Burning | 1.59 ± 0.95 | 1.69 ± 0.86 | 1.00 ± 0.00 | 1.10 ± 0.30 | 1.36 ± 0.79 | 1.45 ± 0.75 |
| Sleepiness | 2.03 ± 1.18 | 2.03 ± 0.97 | 1.67 ± 0.86 | 1.48 ± 0.75 | 1.89 ± 1.07 | 1.81 ± 0.92 |
| Concentration problems | 1.41 ± 0.84 | 1.34 ± 0.75 | 1.33 ± 0.48 | 1.48 ± 0.68 | 1.38 ± 0.71 | 1.40 ± 0.72 |
| Skin redness | 1.03 ± 0.18 | 1.13 ± 0.42 | 1.00 ± 0.00 | 1.05 ± 0.22 | 1.02 ± 0.14 | 1.09 ± 0.35 |

Overview of perceived side effects [mean ± SD] per stimulation condition, following procedure by [38]. Rating scale from 1 (not present) to 4 (severe).
